# iPSC-Derived Microglia as a Model to Study Inflammation in Idiopathic Parkinson’s Disease

**DOI:** 10.3389/fcell.2021.740758

**Published:** 2021-11-05

**Authors:** Katja Badanjak, Patrycja Mulica, Semra Smajic, Sylvie Delcambre, Leon-Charles Tranchevent, Nico Diederich, Thomas Rauen, Jens C. Schwamborn, Enrico Glaab, Sally A. Cowley, Paul M. A. Antony, Sandro L. Pereira, Carmen Venegas, Anne Grünewald

**Affiliations:** ^1^Luxembourg Centre for Systems Biomedicine, University of Luxembourg, Luxembourg, Luxembourg; ^2^Centre Hospitalier de Luxembourg (CHL), Luxembourg, Luxembourg; ^3^Department of Cell and Developmental Biology, Max Planck Institute for Molecular Biomedicine, Münster, Germany; ^4^James Martin Stem Cell Facility, Sir William Dunn School of Pathology, University of Oxford, Oxford, United Kingdom; ^5^Disease Modeling and Screening Platform (DMSP), Luxembourg Institute of Systems Biomedicine, University of Luxembourg and Luxembourg Institute of Health, Luxembourg, Luxembourg; ^6^Institute of Neurogenetics, University of Lübeck, Lübeck, Germany

**Keywords:** microglia, iPSC, neuroinflammation, idiopathic Parkinson’s disease, disease modeling

## Abstract

Parkinson’s disease (PD) is a neurodegenerative disease with unknown cause in the majority of patients, who are therefore considered “idiopathic” (IPD). PD predominantly affects dopaminergic neurons in the substantia nigra pars compacta (SNpc), yet the pathology is not limited to this cell type. Advancing age is considered the main risk factor for the development of IPD and greatly influences the function of microglia, the immune cells of the brain. With increasing age, microglia become dysfunctional and release pro-inflammatory factors into the extracellular space, which promote neuronal cell death. Accordingly, neuroinflammation has also been described as a feature of PD. So far, studies exploring inflammatory pathways in IPD patient samples have primarily focused on blood-derived immune cells or brain sections, but rarely investigated patient microglia *in vitro*. Accordingly, we decided to explore the contribution of microglia to IPD in a comparative manner using, both, iPSC-derived cultures and postmortem tissue. Our meta-analysis of published RNAseq datasets indicated an upregulation of *IL10* and *IL1B* in nigral tissue from IPD patients. We observed increased expression levels of these cytokines in microglia compared to neurons using our single-cell midbrain atlas. Moreover, *IL10* and *IL1B* were upregulated in IPD compared to control microglia. Next, to validate these findings *in vitro*, we generated IPD patient microglia from iPSCs using an established differentiation protocol. IPD microglia were more readily primed as indicated by elevated *IL1B* and *IL10* gene expression and higher mRNA and protein levels of NLRP3 after LPS treatment. In addition, IPD microglia had higher phagocytic capacity under basal conditions—a phenotype that was further exacerbated upon stimulation with LPS, suggesting an aberrant microglial function. Our results demonstrate the significance of microglia as the key player in the neuroinflammation process in IPD. While our study highlights the importance of microglia-mediated inflammatory signaling in IPD, further investigations will be needed to explore particular disease mechanisms in these cells.

## Introduction

Parkinson’s disease (PD) is an age-related, multifactorial disorder, resulting in the demise of dopaminergic neurons in the substantia nigra pars compacta (SNpc) of the midbrain, which subsequently leads to motor difficulties, tremor, and postural instability in affected individuals (Pang et al., 2019). While there is a genetic component to the disease, with 10% of all cases carrying a mutation in one of the causal PD genes, 90% of patients are deemed idiopathic.

The majority of studies published to date describe molecular mechanisms centered around α-synuclein aggregation, mitochondrial dysfunction, dysregulated autophagy flux, and neuroinflammation as the underlying causes of PD (Wang et al., 2015; Maiti et al., 2017). Interestingly, all of these processes are also affected by aging, which leads to functional decline, both at the physiological and molecular level. Thus, it is not surprising that aging is considered a major risk factor for the development of PD (Jin et al., 2020).

Additionally, “inflammaging” is a novel term coined to define basal, low-level inflammation during adult life that, with time, turns into a destructive, pathological process. On the one hand, lower levels of inflammation are considered to have a positive outcome on the overall cellular state. On the other hand, during prolonged inflammation, beneficial mechanisms of defense start to wear off while damaging insults increase. This phenomenon might explain why seemingly low-grade inflammatory occurrences can have a significant negative impact on health in older individuals (Calabrese et al., 2018).

Inflammation is one of the hallmarks of PD and it is propagated mostly through microglia cells, which are responsible for the innate immune defense of the brain. Early brain tissue studies showed an upregulation of microglial cells in the SNpc and higher expression of human major histocompatibility complex class II (MHC-II) molecules, while in human serum and cerebrospinal fluid (CSF), increased concentrations of cytokines such as IL-1β, IL-6, TNF-α, IL-2, IL-18, and, IL-10 were detected (Nagatsu and Sawada, 2005; Brodacki et al., 2008; Long-Smith et al., 2009; Collins et al., 2012; Wang et al., 2015; Badanjak et al., 2021). In line with these results, our own immunohistochemistry and single-nuclei transcriptomic analyses in postmortem midbrain tissue revealed an increase in abundance and a decrease in the complexity of microglia in IPD tissue, suggestive of an activated state. Moreover, patient microglia presented a disease-specific gene expression signature, indicating a significant role of these cells in the pathogenesis of the movement disorder (Smajić et al., 2020). Also, most recently, genes of the IFN-γ signaling pathway were found to be dysregulated in IPD patients (Magalhaes et al., 2021).

One of the most commonly implicated inflammatory pathways in PD is the inflammasome pathway (Chao et al., 2014; Sebastian-Valverde and Pasinetti, 2020; Yan et al., 2020). The NOD-, LRR-, and pyrin domain-containing protein 3 (NLRP3) is by far the most studied inflammasome, the main function of which is the clearance of pathogens. NLRP3 is a cytosolic sensor of intracellular and extracellular stimuli such as damage-associated and pathogen-associated molecular patterns (DAMPs and PAMPs, respectively). Two signals are necessary to fully activate this pathway, a priming signal and an activation signal. The priming signal is characterized by the upregulation of *IL1B* and *NLRP3* expression, while the secondary signal is characterized by the release of mature cytokines (Swanson et al., 2019). Underlining the relevance of the NLRP3 inflammasome, IL-1β has been associated with disease pathogenesis in multiple PD biomarker studies (Koprich et al., 2008; Su et al., 2008; Nakahira et al., 2011; Gillardon et al., 2012; Pike et al., 2021).

In our current study, we investigated inflammation markers in different models of IPD. First, we explored available RNAseq transcriptomic datasets from postmortem midbrain tissues to assess the expression of key cytokines in IPD. Next, we differentiated microglia from iPSC from IPD and control donors to test whether these cells can mirror the phenotypes observed in the brain. We detected elevated levels of *IL1B* and *IL10* in whole tissue or single cell RNAseq datasets from IPD nigral or midbrain sections. Indicative of the fidelity of iPSC-derived cellular PD models, both *IL1B* and *IL10* were also upregulated in patient microglia upon lipopolysaccharide (LPS) treatment. This coincided with increased protein abundance of NLRP3 in these cells, further implicating the inflammasome in the pathogenesis of PD.

## Materials and Methods

### Bulk RNA Cytokine Expression Analysis

To profile the expression of cytokines in human SN tissue, we used a differential expression meta-analysis of publicly available case-control transcriptomics datasets, including only SN samples, as previously described (Glaab and Schneider, 2015). This provided meta-analysis Z-scores and FDR significance scores for candidate genes of interest (see Supplementary Table 1).

### Postmortem Single-Nuclei RNA Sequencing of Human Midbrain

To analyze cytokine expression in a single-cell landscape, in this study, we used our previously published snRNAseq dataset from five IPD and six control postmortem midbrain tissues (GSE157783). The normalization, sample integration and cell clustering were performed using *Seurat* (version 3.1.5) in R 4.0.0., as described in Smajić et al. (2020).

The gene expression analysis was performed in neuronal and microglial clusters derived from our snRNAseq dataset (Smajić et al., 2020). For each of the two clusters, pseudobulk populations were created by merging all cells in the cluster from every individual in order to present the overall expression of cytokines. Then, the expression was presented as a sum of expressions of each individual cell and displayed in a bar plot using *“ggplot2”* and *“gg.gap”* packages. The cytokine expression in microglia was shown in both conditions using the *“DotPlot”* function.

### Differentiation of Human iPSCs Into Microglia

An IPD patient as well as an age- and gender-matched control (both female, age: 68; IPD patient AAO: 60), who donated skin biopsies for the study, gave written and informed consent. Skin fibroblasts were reprogrammed into iPSCs as previously described (Arias-Fuenzalida et al., 2017). The study was approved by the Comité National d’Ethique de Recherche Luxembourg (CNER, vote 201411/05 V1.3). iPSCs were maintained in mTeSR^TM^1 complete medium (StemCell Technologies). Microglia were differentiated from iPSCs following an established protocol (van Wilgenburg et al., 2013; Haenseler et al., 2017). In brief, embryoid bodies (EBs) were generated from iPSCs in mTeSR Plus (STEMCELL Technologies) supplemented with 50 ng/ml BMP-4 (Invitrogen), 50 ng/ml VEGF (Invitrogen) and 20 ng/ml SCF (Miltenyi). On day 4, EBs were transferred to a low attachment 6-well plate and were replenished with fresh EB media. On day 7, the medium was changed to X-VIVO 15 (Lonza) supplemented with 25 ng/ml IL-3 (Invitrogen), 100 ng/ml M-CSF (Invitrogen), 2 mM Glutamax (Gibco), 1% P/S (Gibco) and 0.055 mM β-mercaptoethanol (Gibco) and the EBs were transferred to T75 flasks (factories). These conditions promoted the generation of macrophage precursors. The factories were kept in culture for up to 6–8 months and macrophage precursors were harvested regularly. Terminal differentiation was achieved by culturing macrophage precursors in advanced DMEM/F12 supplemented with N2, Glutamax, P/S, β-mercaptoethanol, 100 ng/ml IL-34 (Peprotech) and 10 ng/ml GM-CSF (Peprotech). During all steps of the differentiation, cells were incubated at 37°C, 5% CO_2_.

### Microglia Treatments

Microglia were seeded into 6-well plates at a density of 1 × 10^6 cells/well. Upon treatment with 100 ng/ml LPS (Thermo Fisher Scientific 00-4976-93) for 3 h, cells were subjected to protein and RNA extractions. For functional analyses, microglia were seeded into 96-well glass-bottom plates at a density of 25,000 cells/well. Cells were treated with 50,000 Zymosan bioparticles (Thermo Fisher Scientific) per well for 45 min. After, cells were subjected to fixation (described in more detail below).

### RNA Isolation and Quantitative PCR

RNA was isolated from microglia using the RNeasy RNA isolation kit (Qiagen, 74106) following the manufacturer’s instructions for direct RNA extraction from the plate. cDNA was synthesized from 200 ng of RNA using the SuperScript^TM^ III Reverse Transcriptase (Invitrogen, 18080044). Quantitative PCR (qPCR) was performed using iQ SYBR Green (Biorad, 170-8885). The PCR reaction was run on a LightCycler 480 (Roche). The samples were denatured for 5 min at 95°C. Amplification ran over 45 cycles with a denaturation step of 10 s at 95°C, primer annealing of 10 s at 60°C, and elongation of 10 s at 75°C. The expression of *IL1B*, *IL10, LRRK2*, and *NLRP3* was normalized to the expression of the housekeeping gene *ACTB*.

### Western Blotting

Total protein from microglia cultures were extracted directly from the plate, using ice cold RIPA buffer (Pierce) supplemented with 1X Protease/phosphatase Inhibitor Cocktail (Thermo Fisher Scientific). The whole well was washed multiple times, on ice, and the lysate suspension was transferred to an Eppendorf tube and vortexed for 20 s followed by incubation on ice for 20 min. The samples were centrifuged at 21,130 g for 20 min at 4°C. The protein concentration of the cell lysates was measured using a bicinchoninic acid assay using Pierce^TM^ BCA protein kit (Thermo Fisher Scientific) following the manufacturer’s instructions.

Cell lysates were denatured in a loading buffer at 95°C for 5 min prior to loading on the gels. Proteins were then separated on NuPAGE 4–12% Bis-Tris gels (Invitrogen) in NuPAGE MES Running Buffer (NP0002) and transferred on a 0.2 μm nitrocellulose membrane. Membranes were blocked with 5% milk in TBS supplemented with Tween-20 (TBST, 10 mM Tris-HCl, 150 mM NaCl, 0.1% Tween-20, pH 8.0) for 1 h at RT. Thereafter, membranes were incubated overnight at 4°C with the following primary antibodies: 1:1,000 anti-NLRP3 (D4D8T, Cell Signaling), 1:500 anti-LRRK2 (75–188, UC Davis), 1:10,000 anti-β-actin (A1978, Sigma). On the next day, membranes were washed three times in TBST and incubated with the respective secondary antibodies for 1 h at RT. Immunoreactivity was detected by enhanced chemiluminescence reaction (ECL select Western blotting detection reagent, GE Healthcare) or near-infrared detection (Odyssey, Li-COR).

### Immunocytochemistry and Image Analysis

IPSCs and microglia were fixed in 4% PFA (Thermo Fisher Scientific, Alfa Aesar J61899) for 15 min and washed twice with PBS (Westburg, LO BE17-513F). The cells were permeabilized and blocked in PBS containing 0.25% Triton X-100 and 1% BSA for 1 h at RT followed by overnight incubation with primary antibodies: anti-Nanog (3580S, Bioke), anti-Sox2 (sc-365823, Santa Cruz), anti-Oct4 (ab19857, Abcam), anti-Iba1 (ab5076, Abcam), anti-P2RY12 (APR-020-F, Alomone labs). On the next day, cells were washed and incubated with the corresponding secondary antibodies. Thereafter, another three washing steps with PBS were completed and Hoechst was used as a counterstain at 0.1 mg/ml for 15 min in PBS. To mount the cover slips onto slides, Prolong Antifade mounting media (Thermo Fisher Scientific) was used. Acquisition of microglia images was performed using a Zeiss LSM 710 and Yokogawa CV8000 microscope, and images of iPSC were acquired with a Zeiss Axio Imager M2. All acquired images were normalized for secondary-only antibody control, to confirm specificity of the signal observed.

For quantitative image analysis, custom code was implemented using MATLAB 2020a, and computations were performed using the High-Performance Computing (HPC) infrastructure of the University of Luxembourg (Varrette et al., 2014). Briefly, the “ZymosanAreaByIba1Area” is the ratio between Zymosan positive pixels and Iba1 positive pixels per field of view. Furthermore, the mean abundance of Iba1 has been quantified as ratio of Iba1 area per nuclei count. The underlying MATLAB code is available upon request.

### Statistics

All experiments carried out using iPSC-derived microglia were performed with 3–4 biological replicates. The data was normalized by the average of values per replicate. For statistical analyses, GraphPad Prism software (version 9) was used. To evaluate the presence of outliers, we used the ROUT test. Two-way ANOVA was used for grouped values. Differences were considered significant (^∗^) when *p*-values were below 0.05.

## Results

### Idiopathic Postmortem Midbrain Tissue Is Exhibiting Increased Cytokine Gene Expression

**FIGURE 1:**
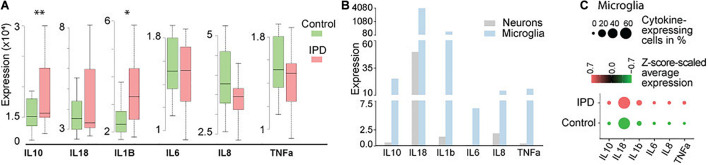
Cytokine expression in bulk RNA-seq and single-nuclei RNA-seq datasets. **(A)** Bulk analysis of SN tissue displays higher expression of *IL1B* and *IL10* in IPD (*FDR* = 0.023; *FDR* = 0.0067). **(B)** Single-nuclei analysis of the human midbrain shows that the overall expression of cytokines is higher in microglia (3903 cells) than in neurons (5314 cells). **(C)** Cytokines are expressed in a large number of IPD and control microglia. Z-score-scaled average expression per cluster shows higher levels in IPD compared to control microglia. “*DotPlot*” function (Seurat package) was used to visualize expression and percentage of cytokine-expressing cells. ^∗^*p* < 0.05; ^∗∗^*p* < 0.01; IPD, idiopathic PD.

Studies implicating inflammatory cytokines in PD have been mostly conducted on neurotoxin and genetic animal models, or by analyzing peripheral blood samples and CSF from PD patients (Badanjak et al., 2021). To further confirm if these findings are indeed occurring in the brain of IPD patients, we analyzed available transcriptomic datasets. The transcriptomics data of human IPD and control SN revealed a significant increase in *IL1B* and *IL10* expression in the patient tissue (*FDR* = 0.023; *FDR* = 0.0067, respectively) (Figure 1A and Supplementary Table 1). A recent study showed that inflammation is not only mediated by microglia but can also be observed in neurons from an IPD mouse model (Panicker et al., 2020). In order to understand whether the detected immune signatures in the human SN are also cell-type specific, we examined our midbrain snRNAseq dataset to obtain an insight into transcriptional changes with single-cell resolution. We confirmed that the expression of cytokines is specific to microglia (Figure 1B). Further analysis of the microglia population revealed higher expression and a larger percentage of expressing cells in IPD compared to control tissue (Figure 1C and Supplementary Table 2).

### Characterization of iPSC-Derived Microglia Model

**FIGURE 2:**
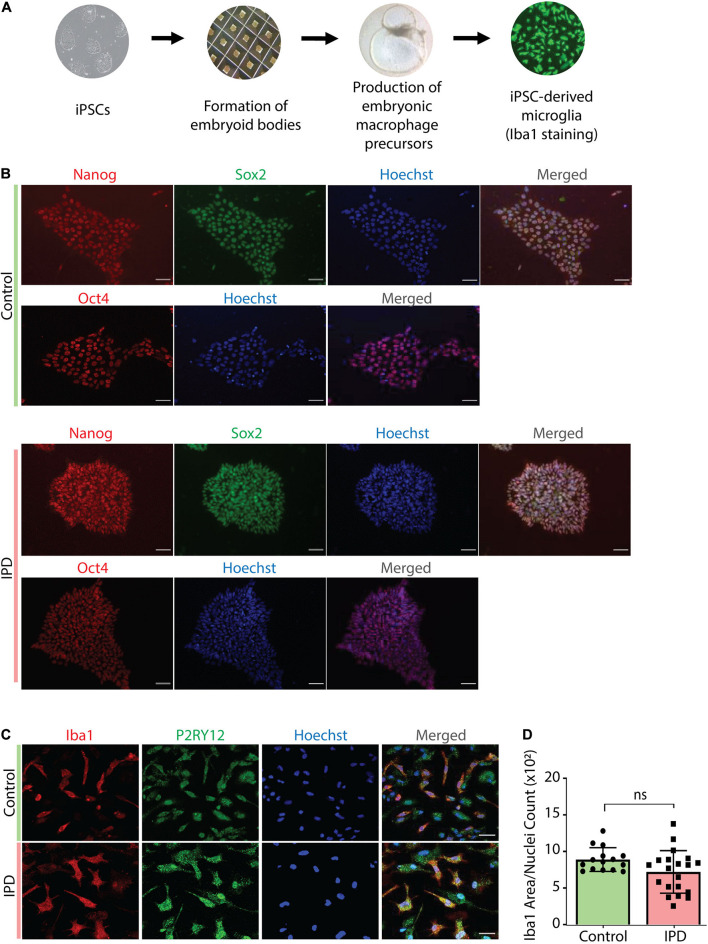
Characterization of cellular models used in the study. **(A)** Overview of the protocol used to derive microglia from iPSCs. **(B)** Immunostaining with Nanog, Sox-2 and Oct-4, coupled with nuclear staining with Hoechst, confirmed the iPSC identity of our cells. Scale bar, 50 μm. **(C)** Terminally differentiated microglia were confirmed to express the microglia-specific markers Iba1 and P2RY12. Scale bar, 30 μm. **(D)** Quantification of data from **(C)**; no difference in Iba1 protein levels (Iba1 area was normalized for nuclei count); IPD, idiopathic PD; CTR, healthy control.

**FIGURE 3:**
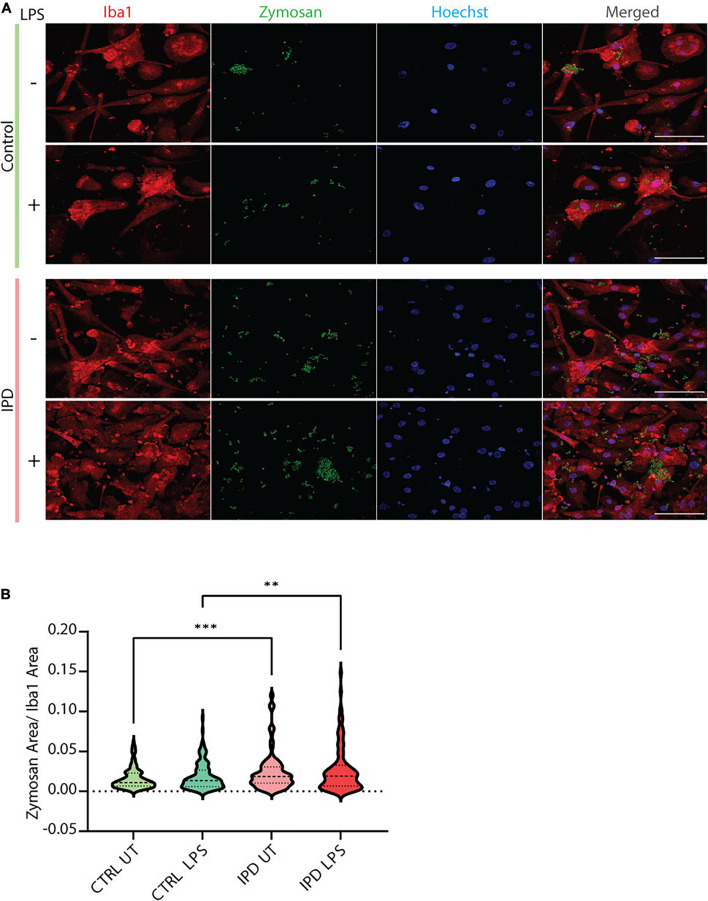
Functional assessment of iPSC-derived microglia. **(A)** Representative immunostaining showing the functional ability of cultured control and IPD microglia treated with Zymosan bioparticles. Scale bar, 50 μm. **(B)** Quantification of data from **(A)**; IPD microglia have higher phagocytic capacity (Zymosan area was normalized for Iba1 area) compared to control cells. ^∗∗^*p* < 0.01; ^∗∗∗^*p* < 0.001; IPD, idiopathic PD; CTRL, healthy control.

IPD patient and control microglia were generated using an established protocol (van Wilgenburg et al., 2013; Haenseler et al., 2017; Figure 2A). The available iPSC lines were characterized by immunostaining with the stem cell markers Nanog, Sox2 and Oct4 (Figure 2B). Differentiation of iPSCs into microglia was achieved with the addition of multiple factors throughout the differentiation process (Figure 2A) to mimic microglia development in the human embryo. The microglial identity of cells was confirmed by positive expression of Iba1 and purinergic receptor (P2RY12) (Figure 2C). Comparable Iba1 areas per nuclei suggest that the disease status of the investigated lines did not have an impact on the differentiation procedure (Figure 2D). Additionally, we wanted to functionally characterize microglia cells by treating them with Zymosan bioparticles and assessing their phagocytic ability. While both control and IPD microglia were able to phagocyte the bioparticles, IPD microglia had a higher capacity (mean: 0.02493, *SD* = 0.02472) compared to control microglia (mean: 0.01606, *SD* = 0.01304; ANOVA: ^∗∗∗^*p* = 0.0004). Furthermore, although the mean phagocytic capacity after LPS treatment was not significantly different between untreated and treated cells, IPD microglia had a significantly higher uptake of Zymosan particles compared to control cells upon addition of LPS (CTR/LPS mean: 0.01897, *SD* = 0.01695; IPD/LPS mean: 0.02683, *SD* = 0.02835; ANOVA: ^∗∗^*p* = 0.0070) (Figure 3).

### Upregulation of NOD-, LRR- and Pyrin Domain-Containing Protein 3 Inflammasome Components in Idiopathic Microglia

**FIGURE 4:**
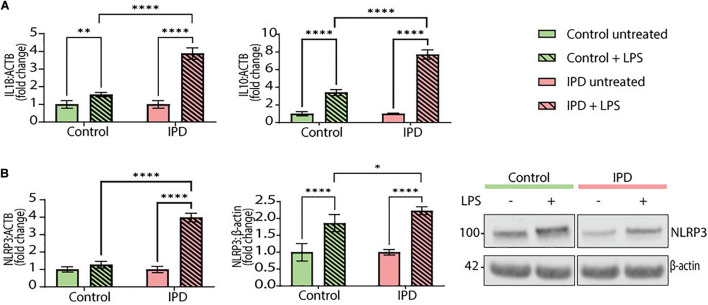
Inflammatory phenotype and LRRK2 levels in iPSC-derived microglia. **(A)** *IL1B* and *IL10* gene expression (normalized to *ACTB*) in control and IPD microglia; a quantitative PCR showed higher gene expressions of *IL1B* and *IL10* in IPD compared to control cells upon priming with LPS (100 ng/ml). Values are represented as fold changes of untreated cells. **(B)** NLRP3 mRNA and protein levels (normalized to *ACTB* and β-actin, respectively) are increased in IPD microglia compared to control cells upon priming with LPS (100 ng/ml). Values are represented as fold change of untreated cells. ^∗^*p* < 0.05; ^∗∗^*p* < 0.01; ^****^p < 0.0001; IPD, idiopathic PD, NLRP3, NOD-, LRR-, and pyrin domain-containing protein 3.

To test the transferability of our findings from postmortem single-nuclei transcriptomics to live cells, we investigated the gene expression of *IL1B* and *IL10* in iPSC-derived IPD microglia. Moreover, we quantified the mRNA and protein levels of the NLRP3 inflammasome, the inflammatory pathway most associated with chronic inflammation in PD. The priming step in inflammasome assembly is frequently mimicked by LPS treatment (McKee and Coll, 2020). At baseline, IPD and control microglia did not show significant differences (data not shown). However, upon LPS treatment, the expression levels of *IL1B* and *IL10* increased significantly in both conditions compared to the respective untreated cells (*IL1B* CTR/LPS fold mean: 1.558, *SD* = 0.117; ANOVA: ^∗∗^*p* = 0.0098; IPD/LPS fold mean: 3.880, *SD* = 0.323; ANOVA: ^****^p < 0.0001; *IL10* CTR/LPS fold mean: 3.409, *SD* = 0.342, ANOVA: ^****^p < 0.0001; IPD/LPS fold mean: 7.717, *SD* = 0.530, ANOVA: ^****^p < 0.0001). When comparing the treatment response between both conditions, IPD microglia showed significantly higher *IL1B* and *IL10* expressions fold changes compared to control cells (*IL1B* LPS mean: 2.719, *SD* = 1.642; ANOVA: ^****^p < 0.0001; *IL10* LPS mean: 5.563, *SD* = 3.047, ANOVA: ^****^p < 0.0001). Additionally, when investigating *NLRP3* expression, only IPD microglia showed a significant upregulation upon LPS treatment, compared to both untreated IPD cells (*NLRP3* IPD/LPS fold mean: 3.984, *SD* = 0.250; ANOVA: ^****^p < 0.0001) and to LPS-treated healthy microglia (*NLRP3* LPS mean: 2.627, *SD* = 1.918; ANOVA: ^****^p < 0.0001) (Figure 4A).

Furthermore, NLRP3 protein levels corroborated the gene expression results. Again, both control and IPD microglia had significantly higher NLRP3 protein levels upon LPS addition compared to their respective basal levels (CTR/LPS fold mean: 1.866, *SD* = 0.254; IPD/LPS fold mean: 2.231, SD = 0.115; ANOVA: ^****^p < 0.0001). However, when comparing across conditions, IPD microglia showed a significant upregulation compared to treated control glia (LPS mean: 2.048, *SD* = 0.2585; ANOVA: ^∗^*p* = 0.0416) (Figure 4B).

### Downregulation of Leucine-Rich Repeat Kinase 2 Upon Lipopolysaccharide Treatment in Idiopathic Microglia

**FIGURE 5:**
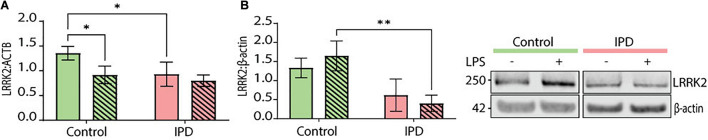
LRRK2 levels in iPSC-derived microglia. **(A)** IPD microglia have a significant decrease of *LRRK2* expression at basal level (normalized to *ACTB*). **(B)** IPD microglia show a significant downregulation of total LRRK2 protein levels (normalized to β-actin) compared to control cells upon LPS treatment only. ^∗^*p* < 0.05; ^∗∗^*p* < 0.01; IPD, idiopathic PD; LRRK2, Leucine-rich repeat kinase 2.

Leucine-rich repeat kinase 2 (LRRK2) is a protein implicated in both the idiopathic and genetic forms of PD. It is highly expressed in cells of the immune system and associated with immune disorders (Van Limbergen et al., 2009; Umeno et al., 2011) as well as infectious diseases (Zhang et al., 2009; Weindel et al., 2020). Its pathogenic effects have been extensively studied in the context of LRRK2-PD and in some instances in IPD. Basal levels of *LRRK2* expression were significantly downregulated in IPD microglia (CTR mean: 1.355, *SD* = 0.136; IPD mean: 0.929, *SD* = 0.244; ANOVA: ^∗^*p* = 0.0226), while control cells had significantly lower expression upon LPS treatment (CTR mean: 1.355, *SD* = 0.136; CTR/LPS mean: 0.915, *SD* = 0.178; ANOVA: ^∗^*p* = 0.0186) (Figure 5A). Furthermore, we investigated LRRK2 protein levels in our iPSC-derived microglia and saw a non-significant downregulation in untreated IPD microglia compared to controls (CTR mean: 1.332, *SD* = 0.257; IPD mean: 0.615, *SD* = 0.425; ANOVA: *p* = 0.0593). Moreover, after stimulating the cells with LPS, IPD microglia had significantly less LRRK2 protein compared to LPS-treated control glia (CTR/LPS mean: 1.649, *SD* = 0.391; IPD/LPS mean: 0.402, *SD* = 0.215; ANOVA: ^∗∗^*p* = 0.0036) (Figure 5B).

## Discussion

Although the majority of PD cases suffer from the idiopathic form of the movement disorder, the cause of neurodegeneration in these individuals has not been extensively investigated. Genome wide association studies (GWAS) identified over 40 PD risk loci, the majority of which overlaps with known autosomal dominant PD genes, most notably *SNCA* and *LRRK2*, while other studies revealed the presence of heterozygous variants in autosomal recessively inherited PD genes (Simón-Sánchez et al., 2009; Nalls et al., 2014; Zeng et al., 2018; Germer et al., 2019; Lai et al., 2020). The main difficulty scientists are facing when studying IPD is the heterogeneous nature of the disease, which is further exacerbated by a plethora of environmental and epigenetic influences.

Inflammation has been considered a hallmark of PD since the late 1980’s, when an upregulation of reactive microglia was first seen in patient brain tissue samples (McGeer et al., 1988). Positron emission tomography (PET) imaging dyes, which allow visualizing activated microglia *in vivo* over time, have been tested as biomarkers for disease progression (Gerhard et al., 2006; Terada et al., 2016; Roussakis and Piccini, 2018). Unfortunately, however, the findings from PET studies have not been successfully transferred to the clinic and the exact molecular mechanisms triggering neuroinflammation in PD currently remain elusive.

Thus, in this study, we investigated patient-derived microglia to explore the inflammatory component of IPD. First, we made use of publicly available transcriptomic data from nigral postmortem tissue to assess the expression of different cytokines in IPD patients compared to age-matched controls. Previous reports established certain secreted cytokines as reliable biomarkers in serum and plasma of PD patients, among them IL-1β, IL-18, IL-6, IL-10, IL-8, TNF-α (Nagatsu et al., 2000; Brodacki et al., 2008; Qin et al., 2016). In line with these studies, our meta-analysis of published brain SNpc case-control transcriptomics datasets indicated elevated levels of *IL10* and *IL1B* in IPD patients.

To explore the cellular origin of this upregulation in IPD, we made use of our previously generated snRNAseq dataset from midbrain IPD and control tissue. Multiple cytokines, including *IL10* and *IL1B*, were predominantly expressed in microglia. This is in accordance with microglia acting as the main player of the immune system in the CNS. Moreover, in the same published dataset, we observed an increase in the microglia number as well as morphological alterations, indicative of an activated state, in IPD patients (Smajić et al., 2020). Next, to corroborate our findings from homogenized SN tissue, we investigated whether any of the aforementioned cytokines show an IPD-specific expression pattern in microglia. While we observed an increase in the expression of all investigated candidates in the patient compared to control cells, the levels of *IL10*, *IL18*, and *IL1B* were the most abundant.

Albeit informative, these postmortem results may be confounded by the fact that they only represent the molecular situation during the latest stage of the disease. Thus, to study inflammatory phenotypes and pathways in an *in vitro* IPD model, we differentiated microglia from control and patient-derived iPSCs using a published protocol (van Wilgenburg et al., 2013; Haenseler et al., 2017). While cultured IPD microglia did not show altered morphology (data not shown), we observed elevated phagocytosis in these cells indicative of overactive immune function. Phagocytosis is an integral part of microglial homeostatic function, and is not only involved in the recognition of self and non-self threats, but also in the engulfment of synaptic elements and the pruning process. Furthermore, enhanced and uncontrolled clearance is contributing to synaptic degeneration. Indeed, multiple PD studies showed loss of presynaptic terminals and synaptic changes in PD patient compared to control brains (Delva et al., 2020; Matuskey et al., 2020). It is also worth noting that disrupting the phagocytic ability of mouse glia was sufficient to rescue the neuronal degeneration phenotype observed in these animals after LPS injection (Bodea et al., 2014). Since we observed the differences in phagocytosis already at basal level, one may speculate that the genetic background in IPD glia contributes to the development of the disease. However, it is still unclear whether overactive, defective or perturbed uptake triggers PD pathogenesis (Janda et al., 2018).

To further validate our findings from postmortem tissue, we analyzed different inflammasome components in the iPSC-derived microglia cultures. IPD microglia were more reactive after priming with LPS, as indicated by enhanced expressions of *IL1B* and *IL10*, and higher mRNA and protein levels of NLRP3 compared to treated control cells. Higher levels of *IL1B* and NLRP3 in IPD microglia indicate a stronger priming step, which is necessary for downstream inflammasome activation and immune response. While we are the first to show NLRP3 dysregulation in iPSC-derived IPD microglia, our results are in agreement with findings from genetic PD models. Specifically, α-synuclein fibrils were shown to induce NLRP3 activation, and loss of the PD-associated protein Parkin triggered the release of mitoDAMPs into the cytosol, which in turn activated the NLRP3 inflammasome in mice (Zhong et al., 2016; Gordon et al., 2018; Ji et al., 2020; Pike et al., 2021). Moreover, NLRP3 was shown to regulate *IL10* levels in mice macrophages, with *IL10* production being decreased in NLRP3^–/–^ mice (Gurung et al., 2015; Kobayashi et al., 2016). This is consistent with our observation of *IL10* and *NLRP3* co-regulation. In line with a biomarker study in serum, IPD patients had higher levels of IL-10 compared to healthy individuals (Rentzos et al., 2009). Furthermore, while the relationship of IL-1β and IL-10 has not been extensively studied in the context of PD, there are reports showing that, under inflammatory conditions, IL-10 selectively inhibits the release of IL-1β (Sun et al., 2019). Patient-derived microglia will be a useful model to explore the molecular mechanisms linking IL-10 and IL-1β in PD in more detail.

Further of interest with regard to inflammation in genetic but also IPD is the kinase LRRK2. Affected individuals harboring mutations in LRRK2 closely mirror the clinical picture of IPD patients (Tolosa et al., 2020) with kinase activity dysregulation being a shared feature of both forms of the disease. Due to its high abundance in immune cells, researchers have speculated that LRRK2 may be crucially involved in the regulation of neuroinflammatory processes (Gardet et al., 2010; Di Maio et al., 2018; Fyfe, 2018). Studies investigating *LRRK2* expression in IPD brain tissue showed a significant downregulation in dopaminergic neurons, which may contribute to the pathology of the movement disorder (Simunovic et al., 2009; Sharma et al., 2011; Yılmazer et al., 2021). In agreement with these reports, we detected significantly reduced *LRRK2* expression and a trend toward lower LRRK2 protein abundance in IPD patient microglia at baseline. Inflammatory insults exacerbated this phenotype, leading to a further reduction in LRRK2 protein levels in the IPD patient-derived cells. However, the exact pathways connecting LRRK2 downregulation to microglia dysfunction in IPD warrant further investigation.

Taken together, inspired by published biomarker studies, we investigated inflammatory phenotypes in different models of IPD. In both, nigral and midbrain RNAseq datasets, we observed a disease-specific upregulation of *IL10* and *IL1B*. Furthermore, from our postmortem single-cell results, we derived that this overexpression predominantly stems from microglia. Next, to test whether we could reproduce this phenotype in a dish, we generated iPSC-derived IPD microglia. Further implicating *IL10* and *IL1B*, in IPD, the expression of these cytokines was also enhanced in patient microglia upon LPS treatment. Finally, we identified an upregulation of NLRP3 on RNA and protein level, corroborating our findings concerning *IL10* and *IL1B*. However, in light of the variability of sporadic PD, our results from a small sample may only be representative for a subset of IPD cases, warranting validation studies in larger cohorts. Moreover, while our study highlights the relevance of microglia in IPD, further experiments will be needed to decipher the exact pathways triggering neuroinflammation in sporadic PD patients.

## Data Availability Statement

The datasets presented in this study can be found in online repositories. The names of the repository/repositories and accession number(s) can be found below: https://www.ncbi.nlm.nih.gov/geo/, GSE157783; https://www.ncbi.nlm.nih.gov/geo/, GSE8397.

## Ethics Statement

Patients gave written and informed consent. The study was approved by the Comité National d’Ethique de Recherche Luxembourg (CNER, vote 201411/05 V1.3).

## Author Contributions

SAC provided training in iPSC-derived microglia. KB, PM, SD, and SLP collected the data. KB, PM, SS, L-CT, PMAA, and EG performed the analysis. KB, PM, SS, PMAA, CV, and AG wrote the manuscript, which was reviewed by all authors. CV and AG conceived the study. ND, TR, and JCS contributed to the establishment of fibroblast cultures and iPSC generation from patient and control cells. AG acquired funding for the study and was in charge of direction and planning of the study.

## Conflict of Interest

The authors declare that the research was conducted in the absence of any commercial or financial relationships that could be construed as a potential conflict of interest.

## Publisher’s Note

All claims expressed in this article are solely those of the authors and do not necessarily represent those of their affiliated organizations, or those of the publisher, the editors and the reviewers. Any product that may be evaluated in this article, or claim that may be made by its manufacturer, is not guaranteed or endorsed by the publisher.
