# Supplementary material for: iPSC-Derived Microglia as a Model to Study Inflammation in Idiopathic Parkinson’s Disease
Source: Front Cell Dev Biol. 2021 Nov 5;9:740758. doi: 10.3389/fcell.2021.740758 (PMC8602578; doi:10.3389/fcell.2021.740758)
Supplement: Supplementary file 1 [file Data_Sheet_1.PDF]

**Supplementary Table 1. Gene expression analysis of human IPD and control SN transcriptome.**

| Gene symbol | Z-score | FDR |
|---|---|---|
| **IL1B** | 2.273739646 | 0.022981645 |
| **IL6** | -0.293008563 | 0.769515607 |
| **IL10** | 2.710259928 | 0.00672305 |
| **IL18** | 1.512416069 | 0.13042805 |
| **IL8** | 1.290107048 | 0.197013493 |
| **TNF** | -0.37382918 | 0.708531408 |

**Supplementary Table 2. Gene expression analysis in IPD and control microglia of the human midbrain single-cell transcriptome.**

| Gene symbol | Average expression | Percent expressed | cluster_id |
|---|---|---|---|
| **IL1B** | 0.325002368 | 5.55967383 | Microglia_IPD |
| **IL6** | 0.003882633 | 0.11119348 | Microglia_IPD |
| **IL18** | 3.170897328 | 63.45441067 | Microglia_IPD |
| **TNF** | 0.011861339 | 0.48183840 | Microglia_IPD |
| **IL10** | 0.015103408 | 0.70422535 | Microglia_IPD |
| **IL8** | 0.014423550 | 0.29651594 | Microglia_IPD |
| **IL1B** | 0.306312685 | 2.98755187 | Microglia_Control |
| **IL6** | 0.002964900 | 0.08298755 | Microglia_Control |
| **IL18** | 3.089786862 | 59.33609959 | Microglia_Control |
| **TNF** | 0.000000000 | 0.00000000 | Microglia_Control |
| **IL10** | 0.007378525 | 0.24896266 | Microglia_Control |
| **IL8** | 0.002578054 | 0.08298755 | Microglia_Control |
